# The human side of automation: experience in clinical pharmacology

**DOI:** 10.1155/S1463924695000162

**Published:** 1995

**Authors:** J. Robert Powell

**Affiliations:** Clinical Pharmacology Glaxo Research Institute Research Triangle Park NC 27709 USA


Journal of Automatic Chemistry, Vol. 17, No. 3 (May–June 1995), pp. 95–98

### The human side of automation: experience in clinical pharmacology

**J. Robert Powell**
*Clinical Pharmacology, Glaxo Research Institute, Research Triangle Park, NC 27709, USA*

A vision of automation presented by the media is that robots are inherently smarter than humans. Robots whirl around efficiently doing a complex, tedious task. A single human monitors and programs many robots and markedly increases productivity and quality, while many previously employed, error-prone employees collect their unemployment benefit.

My experience of automation from an industrial clinical pharmacology department is quite different from this. In an environment where workload and complexity increase progressively in the face of a fixed human and financial resource, seeking efficiency through automation has been synonymous with success, if not survival. In addition to using robots to automate physical processes, we automate information with computers and standardize repetitive, labour intensive tasks with more efficient processes. Direct by-products of the increased productivity through automation are enhanced creativity and job satisfaction. The irony to me is that automation is by its nature, very human.

Before I can describe automation in my environment I have to explain the nature of our work and the challenges we face.

Glaxo has been a rapidly growing company for the past 15 years. The current clinical pharmacology department had its origins when a bioanalytical group was formed 10 years ago to service clinical studies. Seven years ago, pharmacokineticists were hired to service new formulations being developed for UK discovery drugs. Three years ago our mission expanded to bring new chemical entities into human phase I studies. The mission is now to advance or stop drugs from progression to phase II patient efficacy studies, to recommend dosage regimens for the primary patient population and special groups (for example children), and to understand how drugs act in humans.

To accomplish this mission we have assembled a diverse group (about 40 people) of scientists and support staff including analytical chemists, pharmacokineticists, specialty trained physicians, clinical monitors, computer programmers, and administrative staff skilled in computer graphics and information processing.

In general terms, our scientific focus is biopharmaceutics, pharmacokinetics, and pharmacodynamics. These principles are applied to a diverse group of therapeutic targets: bacterial and viral infection, cancer, diabetes, obesity, benign prostatic hypertrophy, hypertension, asthema, cystic fibrosis, migraine, ulcers, stroke, and nausea. The mix of projects and problems changes very rapidly, requiring rapid access to information, clear decision points, and staff flexibility.

In our drug development environment, clinical pharmacology is the customer of preclinical development in pharmacology, toxicology, drug metabolism and pharmaceutics. We work with drug discovery and preclinical development to provide a clear phase I target for patient type, human dosage range estimate and route of administration.

Clinical pharmacology customers are the phase II/III and IV therapeutic groups in gastrointestinal, cardiovascular, infectious, central nervous systems, cancer, and respiratory diseases. We provide these groups an early estimate for drug safety, therapeutic activity, and dosage recommendations. International co-ordination is required between clinical pharmacology groups in England, Italy, Canada, France, and the USA.

The overall complexity and required speed of our work is further challenged by the current re-engineering targets to increase productivity several fold and decrease our development and FDA drug approval times.

I hope this background picture demonstrates the acute need for efficiency in planning, research execution, decision making and flexibility to recycle our resource as needs change. Automation is not an interesting experiment . . . it is central to our success!

Before I introduce automation examples, I want to describe a few of our human environment goals:

(1) We assume people have an inherent desire to work, that, when well managed and led, they will use their energy and imagination to achieve their personal goals and thereby contribute to the organization. Parenthetically, this most important of all assumptions (Theory Y) was described in *The Human Side of Enterprise* by Douglas McGregor in 1960 from which the title of this paper was paraphrased. The opposite assumption is Theory X which assumes people dislike work and must be driven to perform.
(2) Hire smart, well-trained people with 'fire in the belly'.
(3) Provide clear department and individual planning.
(4) Reinforce a customer focus through project management and personal assessment.
(5) Develop people and facilitate their growth inside and outside the company.
(6) Strive for accountability of teams and individuals.
(7) Maintain good stress, prevent or rapidly eliminate destructive stress.
(8) 'Kick up/kiss down'. This phrase, coined by a friend, captures a fundamental need for my environment. Managers need to have the freedom to deliver unpopular information to more senior management.

© Zymark Corporation 1995

Putting 'spin' on critical information can literally kill a company. Fear of senior management is fundamentally unhealthy.

(9) Work is a contact sport which should be fun on most days.

(10) Be committed! As with eggs and bacon for breakfast, the chicken was involved, but the pig was committed.

The three automation types we employ are mechanical, information/computer, and process.

### Mechanical automation

Having formed a bioanalytical group in 1985 to service clinical assay demands, the demand and time limits required for service were frequently greater than capacity. By 1988, this productivity issue and the knowledge that we would be developing an AIDS drug (need to limit employee exposure) stimulated the laboratory manager to begin a robot pilot study employing Zymark equipment. Within nine months we saw this was going to be successful and we began hiring additional expertise and expanding to the current five units. Continued analytical demand and analyte diversity have led us to contract out about one-half the total samples per year.

Developing internal robotics and an innovative external contracting service has allowed us to develop a different bioanalytical philosophy which supports our mission and customer focus. We begin developing our assays six months prior to the first human studies with an automation target. Our first studies in humans are designed to yield safety and pharmacokinetic information. As the dose is increased, at about weekly intervals, we collect the blood and urine for immediate shipment back to the Glaxo bioanalytical laboratory. Bioanalytical results are usually given to the project leader within days of sample receipt and before the next dose escalation is begun. This allows the project leader to consider human drug exposure in relation to the clinical effects being observed. This can be compared to similar observations in the animal toxicology studies.

We operate our drug assay lab at this development point much as one would a clinical laboratory in the hospital. If the samples were batched until the end of the study as has been traditionally done, we would not learn as much and adapt while the protocol is in progress.

Mechanical robots have been essential to developing this level of service. Efficiency gained through robotics then pointed to a new bottleneck in chromatographic information processing, which is addressed in the second example.

Bioanalytical robotics have resulted in benefits for the company and people:

*Company*

(1) Productivity increased about three fold.
(2) The technology has spread to the UK for similar clinical applications and to drug metabolism for toxicology support.
(3) Assay of the same analyte at two sites (US, UK) using the same equipment is more uniform.

*People*

(1) Job paradigm shifted from mechanical to thinking. Tying a chemist to a machine where repetitive extractions and sample injections are performed can become mind-numbing when done day after day, year after year. This had led to turnover.
(2) Morale markedly improved.
(3) Creativity was stimulated since there was more time to think.
(4) People enjoyed the increased productivity by working smarter, not faster and longer.
(5) Chemist visibility and importance to the project team increased since they were delivering key information when decisions could be made.
(6) Pay increased.

Now the laboratory seems to be shifting from conventional HPLC to LC/MS technology. We expect robotics to continue while adapting to the new need.

### Information/computer automation

Early in forming the department we decided to automate the information flow from clinical study protocol initiation to final report submission (see figure 1). Together with colleagues from the Information Technology Department, we created a master plan in 1988 and have been generating new modules ever since. Our goal was to create speed and efficiency by standardizing our processes in user friendly software. More recently we joined forces with our UK colleagues to create a common information highway, language, methods, and standards within the company. Global information standardization is a fundamental need for an integrated global research company. Without this, communication is impaired and people are allowed to differ unnecessarily. Time is wasted and quality is decreased.

For a new chemical entity being given to humans for the first time, the protocol is planned and the study is performed. Safety and pharmacodynamic data are returned to the company for storage in a clinical data base. Blood and urine samples are returned to the laboratory for analysis. Within the lab an analytical sequence is set and an electronic spreadsheet is set to receive the final sample concentrations. Before the

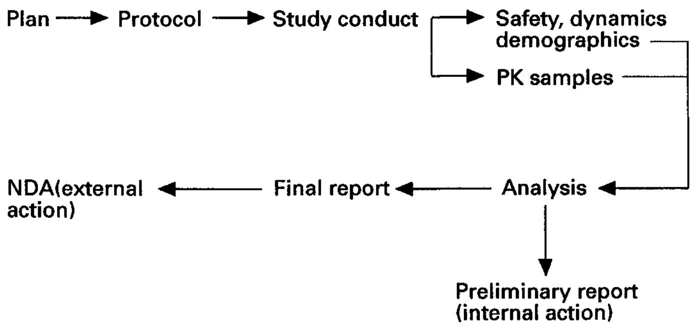

*Figure 1. Information flow data.*

*Table 1. Clinical pharmacology information system.*

| | |
|---|---|
| Clinical pharmacology planning<br>■ Study information/analytical research<br>■ Date milestones<br>■ Site information<br>■ Value added<br>Resource allocation<br>Labels<br>Time—communication savings: 3–6 days/study | Sample management<br>■ Initiate, track, report specimens<br>GLP/GCP compliant<br>■ Spreadsheet generation—SOP<br>■ Links<br>Protocol to data analysis<br>PK to clinical information<br>■ Value added<br>Quality—built in QA<br>Time savings: 1–2 weeks/study |
| Sample sequence generator<br>■ Analysis order:<br>■ Value added<br>Sample tracking<br>Time savings: 1·5 days/study | LAS<br>(Laboratory Automation Systems)<br>■ HPLC integrator<br>■ Value added<br>Quality and consistency<br>Security<br>Documentation<br>Time savings: 2–4 weeks/study |
| PRANBAS<br>(Postrun analysis of bioanalytical samples)<br>■ Standard curve<br>—regression—7 models<br>■ QC samples<br>■ Spreadsheet population and management<br>■ Bioanalytical figures and tables—report<br>■ Value added<br>Compliance with SOPs<br>Time savings: 3 weeks | PK and PD Analysis<br>■ Toolbox menu<br>■ Method sheets—international<br>Bioequivalence<br>Dose proportionality<br>Drug interactions<br>Food<br>Renal<br>Hepatic<br>■ Clinical pharmacology report—figures and tables<br>■ Value added<br>Quality and consistency<br>Security<br>Documentation<br>Time savings: 2–4 weeks/study |

concentrations are known the chromatograms are electronically integrated and a post-run analysis is conducted. The final sample concentrations then populate the electronic spreadsheet at which point the data are released from the chemist to the pharmacokineticist or statistician. At this point the data are available for pharmacokinetic analysis, or they can be merged with dynamic data from the clinical database for a simultaneous kinetic/dynamic analysis. For routine data analyses (for example bioequivalence, dose proportionality, drug interactions, renal failure), we have internationally agreed to uniform method sheets for data analysis and presentation. The system and components are outlined in table 1.

The benefits from continuing to build this system include:

(1) Time savings of about three person months per study. We currently conduct about 40 US studies a year, so this saves about 120 person months yearly or the equivalent of 10 people. Savings will expand as the system is implemented internationally.
(2) Standardization: by deciding how we wish to do our work ahead of time, we avoid asking people to repetitively solve the same problem, arriving at different solutions for the same problem. Hence, our reports are written to an international standard as opposed to local standards.
(3) Ease of data access and movement. Historically, the pharmacokineticist or statistician may spend 10-fold more time getting a data-base set up than the actual data analysis time.
(4) Speed in report generation. Preliminary reports are written within hours of data availability, while final reports are expected within 90 to 120 day of the last patient studied.
(5) Fewer errors, since data are transferred electronically and data analysis uses pre-approved methods.

(6) Resource planning is improved since key study milestone dates are shared amongst the project leader, chemist, pharmacokineticist, data management specialist, biostatistician, and project planner. Changes in dates or priority allow rapid resource adjustment.

The current plan is to extend this project by automating the report into the electronic New Drug Application.

### Process automation

A different example is automating expense reports generated from travel. Our department spends much time travelling to clinical study sites and meetings around the world. Historically, obtaining reimbursement in time to pay the credit card bills has induced stress. Several administrative assistants collaborated to create a simple system for their customers (the traveller) which increases the ease of expense documentation, reduces calculation errors, and speeds reimbursement. The traveller is supplied with a letter-size envelope when they receive their airline tickets. They are requested to drop receipts into the envelope and record them on a daily spreadsheet printed on the envelope front surface. Upon return they hand the filled envelope to the administrative assistant who then keys the information into an electronic spreadsheet, which automatically calculates currency exchanges and yields totals. Reimbursement occurs within a few days. The benefits of this system are ease of use, decreased calculation errors, and standardized presentation to accounting. All this means that people are reimbursed more rapidly with fewer hassles. As this simple automated system is spreading across the company, it is estimated that 5–10 positions may be saved. Service has been improved and people can spend their time more productively on our primary business—discovering, developing, and selling drugs.

Automation is a mind-set which allows chemists, pharmacokineticists, physicians, clinical monitors, and secretaries to perform their jobs more efficiently. In my environment automation has both given people a paradigm for problem-solving, as well as freeing them to more creatively solve their work problems. Like the automated systems and robots we use in clinical pharmacology, I know that inside my first 1950s television robot, Tobar, on *Captain Video* and my last movie robot in *The Terminator*, a human being was performing inside the robot shell.

### Acknowledgments

*Mechanical robots:* Julie Tomlinson, Tom Lloyd, Ron Lang, Moira Dunne; *information computers:* Keith Muir, Jim Johnson, Alan Bye, Karl Donn; and *process:* Dianne Calvert, Wendy Anders and Paul Rubin.

